# A Rare Case of Varicella-Zoster Virus Encephalitis Presenting With Lost Ability to Play the Piano in an Immunocompetent Pediatric Patient

**DOI:** 10.7759/cureus.41383

**Published:** 2023-07-05

**Authors:** Nancy Le, Daniel I Razick, Anand Dhaliwal, Muzammil Akhtar, Emily Daniel

**Affiliations:** 1 Neurology, California Northstate University College of Medicine, Elk Grove, USA; 2 Surgery, California Northstate University College of Medicine, Elk Grove, USA; 3 Pediatrics, Sutter Health, Sacramento, USA

**Keywords:** immunocompetent children, vzv encephalitis, clinical virology, neurology, pediatrics, altered mental state, varicella encephalitis, varicella-zoster virus

## Abstract

Varicella-zoster virus (VZV) is a member of the alpha-herpesvirus family, which can occasionally cause severe neurological complications such as encephalitis. In this case report, we discuss a rare finding of VZV encephalitis in which an immunocompetent pediatric patient, vaccinated against varicella, presented with altered mental status and no vesicular rash. A 15-year-old male presented to the Emergency Department with progressively worsening altered mental status over the past three days. The patient's mother stated that he was exhibiting frequent memory lapses as well as the sudden loss of the ability to play the piano. After admission to the pediatric general floor, lumbar puncture was performed and cerebrospinal fluid analysis returned positive for VZV, confirmed by polymerase chain reaction. The patient was then started on intravenous (IV) acyclovir at a dose of 650 mg every 8 hours to treat VZV-induced encephalitis. While the patient continued to have intermittent episodes of confusion and headaches, his overall condition improved, and by day 4, he was able to resume playing the piano and ukulele. The patient was discharged on day 8 with no home medications, and a follow-up with this primary care physician was scheduled. This patient is one of only four recorded cases of VZV encephalitis in immunocompetent children. It is extremely rare to encounter pediatric patients with this diagnosis and, as such, can elude physicians when developing differential diagnoses. If VZV is suspected, a lumbar puncture should be performed promptly, and, if confirmed, IV acyclovir should be started. Furthermore, this case highlights the need for future research with regard to VZV and potential predisposing factors in immunocompetent patients.

## Introduction

Varicella-zoster virus (VZV) is an exclusively human member of the alpha-herpesvirus family and is the causative agent of varicella and zoster. Upon initial infection, which typically occurs in childhood, the virus causes varicella and then becomes latent in the dorsal root and cranial nerve ganglia. Adverse effects are commonly seen in reactivation, which results in zoster [1]. While the majority of cases are benign and self-limiting, VZV can cause neurological complications such as encephalitis. VZV encephalitis, which is a rare but deadly consequence of VZV infection, can cause long-term neurological impairments or even death [2]. Encephalitis has been reported to occur in one out of every 33,000 to 50,000 cases, according to the World Health Organization (WHO), with the highest incidence occurring in elderly and immunocompromised individuals [3].

VZV encephalitis typically presents as fever, headache, and altered mental status, which can rapidly progress to focal neurological deficits, seizures, and coma. Vesicular rash in one or more dermatomes is often noted preceding or following other manifestations. Diagnosis is made primarily by clinical suspicion and confirmed by polymerase chain reaction testing [4]. While intravenous acyclovir is the recommended treatment, optimal duration of treatment and the role of adjunctive corticosteroid use remain unstandardized [5]. Early diagnosis and timely treatment are essential for improving clinical outcomes in VZV encephalitis cases, as demonstrated in the discussed patient. We present a rare case of pediatric VZV encephalitis in an immunocompetent, vaccinated individual.

## Case presentation

A 15-year-old immunocompetent male with a history of migraines, anxiety, Tourette’s syndrome, and attention-deficit/hyperactivity disorder (ADHD) presented to the Emergency Department (ED) with progressively worsening altered mental status, which began three days prior. Per the patient’s mother, the patient started exhibiting frequent 10- to 15-second memory lapses as well as sudden loss of the ability to play the piano despite previous piano proficiency. He had been very frustrated and agitated the last few days, unlike his usual demeanor.

Upon arrival, the patient’s vital signs were as follows: blood pressure of 125/69, heart rate of 54 beats per minute, temperature of 97.9 degrees Fahrenheit, respiratory rate of 18 breaths per minute, and SpO_2_ 100% on room air. The patient weighed 66 kilograms (145 lbs 8.1 oz), and his height was not recorded. Physical examination did not reveal diffuse skin changes or rashes. No fever was noted. Toxicity screening, complete blood count, comprehensive metabolic panel, and chest X-ray were unremarkable. The patient was vaccinated against varicella at three months of age and again at two years. Pediatric neurology was consulted, and the patient was admitted to the pediatric unit for further evaluation

On the pediatrics floor, the patient was able to communicate and follow commands. He stated that his head “felt like numbness.” Evaluation by a pediatric neurologist and review of systems revealed rhinorrhea, positive change in activity, headaches, agitation, confusion, and decreased concentration. Notable negative findings included seizures, dizziness, weakness, or speech difficulty. Neurological examination was positive for forgetting the date and agitation. Extraocular movements and gait were normal. MRI without contrast did not reveal acute infarction, mass, hemorrhage, or midline shift. Ventricles were normal, and cerebellar tonsils were in the normal position. The patient was then admitted to the pediatric general floor. After admission, a repeat MRI was performed and found to be unremarkable. However, lumbar puncture (LP) was performed followed by cytology which was positive for VZV. Viral polymerase chain reaction (PCR) confirmed VZV infection. Cerebrospinal fluid (CSF) chemistry analysis showed red blood cells, an abnormal finding, whereas total proteins and glucose were within the normal range (Table 1). These findings are consistent with a viral infection as opposed to a bacterial infection.

**Table 1:** Patient’s CSF findings CSF, cerebrospinal fluid

	Patient lab values	Reference range
Red blood cells (cumm)	1	0
Total proteins (mg/dL)	29	15-60
Glucose (mg/dL)	57	50-100

The patient was then started on intravenous (IV) acyclovir at a dose of 650 mg every 8 hours to treat ZV-induced encephalitis. After acyclovir treatment was started, CSF gram stain was performed to rule out bacterial infection, which returned negative. Rapid improvement in symptoms was noted, and IV acyclovir was continued for six more days. On day 3, repeat PCR confirmed VZV, and repeat CSF analysis was performed to rule out concomitant herpes simplex virus (HSV) or enterovirus infection. Analysis revealed sole VZV infection. The patient continued to have intermittent episodes of confusion and headaches during admission, but his overall condition improved, and by day 4, he was able to resume playing the piano and ukulele. By day 5, his piano playing skills had returned to baseline proficiency and he was able to play complex pieces without issue. He was discharged on day 8 following completion of the seven-day course of acyclovir. No home medications were prescribed, and a follow-up with the patient's primary care physician was scheduled.

## Discussion

VZV encephalitis typically presents with delirium following emergence of vesicular rash [4]. Symptoms found in viral encephalitis patients classically include fever, headache, seizures, and altered mental status. Behavioral changes, hallucinations, and cognitive decline are also frequently seen [6]. Rash was absent in this patient prior to, during, and following the diagnosis of VZV. He was also afebrile and did not present with seizures. Altered mental status, behavioral changes, and cognitive decline in the form of inability to play the piano were consistent with typical findings. Notably, VZV encephalitis most commonly affects immunocompromised and elderly patients (age 60 and above) [4]. This patient is one of only four recorded cases of VZV encephalitis in immunocompetent children. Compared to the other three patients, our patient did not present with a fever, had unremarkable MRI and EEG, was given acyclovir IV for seven days compared to 14, and had no sequelae (Table 2).

**Table 2:** Comparison of four recorded cases of pediatric VZV encephalitis Ab, antibodies; CSF, cerebrospinal fluid; DNA, deoxyribonucleic acid; EEG, electroencephalography; HIV, human immunodeficiency virus; Ig, immunoglobulin; IV, intravenous; MHC, major histocompatibility complex; MRI, magnetic resonance imaging; NK, natural killer; PCR, polymerase chain reaction; T, temperature; VZV, varicella zoster virus

	Chiappini et al., 2002 [7]	Spiegel et al., 2010 [8]	Ciancia et al., 2020 [9]	Our patient, 2023
Age and gender	2-year-old boy	14-year-old girl	12-year-old girl	15-year-old boy
Chickenpox/vaccination (age)	Chickenpox (4 months)	Chickenpox (4 years)	Chickenpox (NA)	Chickenpox (3 months and 2 years)
Previous reactivation	No	Zoster (10 years)	No	No
Clinical presentation:				
-Fever	Yes (T: 38.5°C)	Yes (T: 39.2°C)	Yes (T: 38°C)	No (T: 36.6°C)
-Neurological symptoms and signs	Frontal headache, vomiting, disturbed consciousness, miotic pupils, tendon reflexes absent, left ankle clonus, bilateral Babinski’s reflexes elicited	Paresthesias and hyperesthesia in the left limbs, proximal left leg weakness, severe frontal and occipital headache, stiff neck, positive Brudjinski sign	Severe frontal headache, vomiting, photophobia, altered mental status, psychomotor agitation	Altered mental status, headache, cognitive decline, agitation, memory loss, confusion
-Skin rash	No	No	No	No
CSF:				
-White cells/mL; reference range (0-5)	Normal	434	484	Normal
-Protein (mg/dL); reference range (15-60)	Normal	59	72	29
-Glucose (mg/dL); reference range (50-80)	Normal	49	52	57
-PCR for VZV-DNA	Positive	Positive	Positive	Positive
EEG	Findings compatible with viral encephalitis	Right-hand-sided slow waves, compatible with right hemisphere encephalopathy	Severe alteration of cortical electrogenesis with exacerbation of diffuse slow cortical activity, with fronto-temporal predominance	Unremarkable
MRI	Bilateral multifocal changes in white and gray matter, predominantly on the fronto-parietal cortex	Four hyperintense lesions without enhancement after gadolinium injection: 1 in the right thalamus, 1 in the right temporal subcortical region, and 2 in the right parietal subcortical region	Unremarkable	Unremarkable
Immunological screening	HIV Ab negative; IgG, IgA, IgM serum levels, B- and T-lymphocyte counts, NK cell subset counts, in vitro T-lymphocyte response to mitogens, MHC I and II class molecule-positive cell counts: normal	HIV Ab negative; IgG, IgA, IgM serum levels, complement studies, total counts, and functional studies of T cells: normal	HIV Ab negative; IgG, IgA, IgM serum levels, B- and T-lymphocyte counts	HIV Ab negative; IgG, IgA, IgM serum levels, B- and T-lymphocyte counts normal
Treatment (days)	Acyclovir IV (15 days); ceftriaxone IV (5 days)	Acyclovir IV (14 days); methylprednisolone IV (5 days)	Acyclovir IV (14 days) followed by oral acyclovir (14 days)	Acyclovir IV (7 days)
Sequelae	Mild right hemiparesis	Left thigh neuralgia	No	No

Acute and subacute complications associated with VZV are numerous. However, long-term effects still warrant investigation. One patient was recorded to develop behavioral tics and attention deficit disorder following VZV encephalitis infection [10].

VZV is a highly infectious virus encountered worldwide, resulting in a plethora of manifestations. Primary infection, known as varicella or “chickenpox,” typically presents with fever, chills, night sweats, and a widely disseminated pruritic and vesicular, papular, pustular, or papulo-pustular rash involving the face and trunk [11]. VZV infects the nasopharyngeal lymphoid tissue through airborne droplets during early stages of varicella infection, which results in a viremia of infected cells ultimately traveling throughout the rest of the body [12]. The virus partially evades immune responses by downregulating major histocompatibility complex (MHC) class I and inhibiting interferon response genes [13]. Reactivation of the virus produces herpes zoster (HZ). This results in inflammation of the involved sensory ganglion as well as hemorrhagic necrosis of nerve cells, which causes typically associated neuropathic pain [14]. The incidence of HZ has been estimated to be 1.2 million in the United States alone, with incidence rates progressively increasing with age. Not only is the incidence higher in older populations, but severity of disease and likelihood of complications has also been shown to increase with age. Immunocompromised patients are at an elevated risk of HZ due to reduced T-cell mediated immunity, with rate of complications also significantly higher in this patient population [15].

VZV is associated with many neurological manifestations beyond acute neuritis associated with the common pruritic lesions. Postherpetic neuralgia (PHN) is defined as significant pain persisting for 90 days following the onset of rash. Numbness, dysesthesias, pruritus, and allodynia in the affected dermatome are common associated symptoms. Individuals 60 years and older account for half of PHN cases [16]. Ramsay Hunt syndrome, also known as herpes zoster oticus, is another manifestation of VZV and presents as a triad of ipsilateral facial paralysis, vesicles in the auditory canal, and ear pain [17]. Patients with HZ have been reported to develop aseptic meningitis with LP revealing CSF pleocytosis and rash at the time of diagnosis [18]. VZV vasculopathy can produce stroke syndromes secondary to cerebral artery infection as evidenced by a case series conducted by Nagel et al. [19]. Peripheral motor neuropathy develops in approximately 3% of patients with HZ as a result of VZV spreading to the anterior horn of the spinal cord [4]. Patients with a recent history of HZ have also been found to have an increased risk of developing Guillain-Barré syndrome [20]. VZV has also been commonly found to cause both varicella pneumonia and pneumonitis, particularly in immunocompromised patients [21]. However, VZV infection and clinical manifestations have been rarely reported in immunocompetent patients. Given this, further research is prompted regarding potential predisposing factors in immunocompetent hosts.

This case report has its limitations. Given the rare nature of this presentation, it is difficult to generalize the findings of the report. The patient in this case was vaccinated and immunocompetent, which makes establishing a causal relationship between his VZV infection and his symptoms complicated. However, since the acyclovir treatment resolved the symptoms, it is likely that VZV was the underlying cause.

## Conclusions

VZV can manifest in several ways, with encephalitis being one of the more severe consequences. Though rare, VZV encephalitis can be deadly if not diagnosed and treated swiftly. With this case report, the goal is to present an unusual case of VZV encephalitis in an immunocompetent pediatric patient with a history of Tourette’s syndrome. It is extremely rare to encounter pediatric patients with this diagnosis and, as such, can elude physicians when developing differential diagnoses. If a patient presents with altered mental status with no recent drug or alcohol intoxication, an LP should be performed promptly to rule out encephalitis. If a diagnosis of VZV encephalitis is made, IV acyclovir should be administered for a minimum of seven days. Furthermore, this case highlights the need for future research with regard to VZV and potential predisposing factors in immunocompetent patients.
